# Iodine Rearrangements of Tetraallylsilane and Synthesis of Silicon-Stereogenic Organosilanes

**DOI:** 10.3390/ijms25189996

**Published:** 2024-09-17

**Authors:** Elliott D. Tan, Kerry E. Wier, Gregory W. O’Neil

**Affiliations:** Department of Chemistry, Western Washington University, Bellingham, WA 98225, USAwierk@wwu.edu (K.E.W.)

**Keywords:** allylsilane, rearrangement, ring-closing metathesis, silacycle, stereogenic silicon

## Abstract

Tetraallylsilane can undergo either a mono or double rearrangement when treated with iodine (I_2_). The extent of rearrangement depends on the equivalents of I_2_ used, where 1 equivalent gives high yields of mono-rearranged products and excess (e.g., 3 equivalents) causes double rearrangement to occur. This transformation can be applied to the synthesis of potentially valuable silicon-stereogenic organosilanes.

## 1. Introduction

**Scheme 1:**
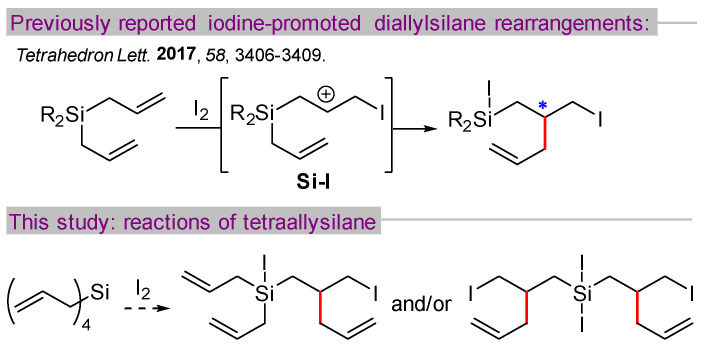
Iodine-promoted rearrangement of diallylsilanes [1].

During the course of investigating iodine-catalyzed etherification reactions of allylsilanes, our group discovered an iodine-promoted rearrangement of diallylsilanes (Scheme 1) [1]. The reaction is thought to proceed through a mechanism involving the intramolecular allylation of a beta-silyl carbocation **Si-I**, generating a new carbon–carbon bond (highlighted in red) and a new stereocenter (*). Several different diallylsilanes with differing substitutions at silicon (i.e., Me_2_, i-Pr_2_, Ph_2_, cyclotrimethylene) were effectively engaged in this transformation, with diallyldiphenylsilane giving the best ratio of rearrangement versus competing iododeallylation. 

We questioned how tetraallylsilane might behave in this reaction, presenting not only an opportunity for a double rearrangement process but also perhaps an increased likelihood of competing side reactions (e.g., deallylation [2] or polymerization [3]). In this paper, we report results from reactions of tetraallylsilane with iodine, demonstrating the ability to select for mono- or double-rearrangement products in good yield. Additionally, this strategy was used to generate potentially valuable silacyclic compounds containing a stereogenic silicon atom.

## 2. Results and Discussion

**Figure 1:**
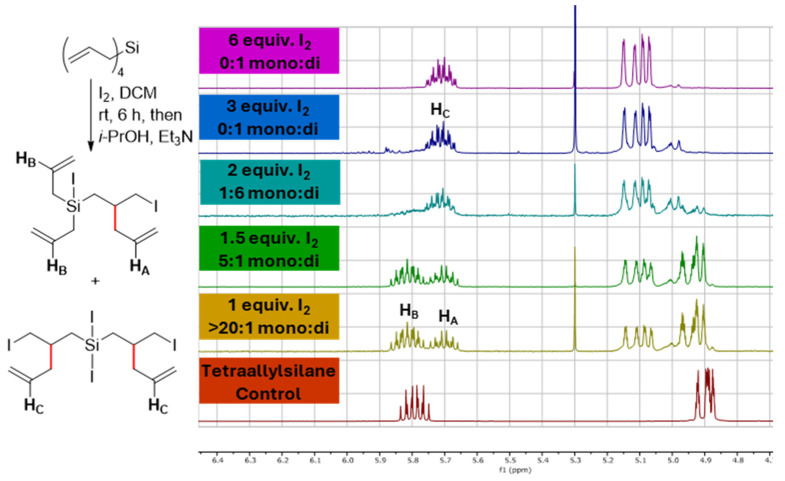
NMR analysis of tetraallylsilane reactions with differing equivalents of iodine (I_2_) showing that good selectivity for the single (mono) and double (di)-rearranged products could be obtained by controlling the amount of I_2_ used.

To begin our investigation, tetraallylsilane was treated with different equivalents of I_2_ using CD_2_Cl_2_ as the solvent, allowing for direct product analysis with NMR (Figure 1). As shown in Figure 1, using 1.0 equiv. of I_2_ resulted in the nearly complete consumption of the starting material and the selective formation of the mono-rearranged product. Increasing the amount of I_2_ to 2 equivalents then caused the di-rearranged product to predominate.

**Scheme 2:**
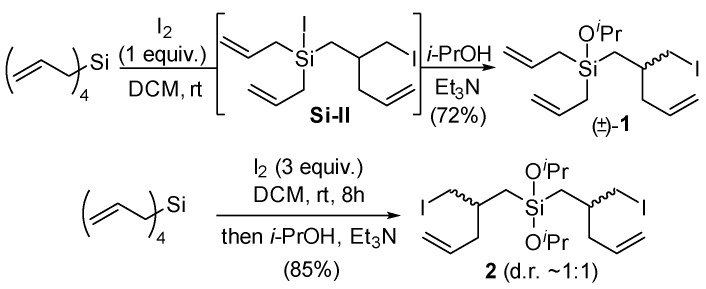
Synthesis of **1** and **2** from tetraallylsilane by iodine-promoted rearrangement.

From the equivalents study, 1.0 equiv. of I_2_ was selected as optimal for the mono rearrangement of tetraallylsilane, and 3.0 equiv. of I_2_ was selected for the double rearrangement. In this way, compounds **1** and **2** were obtained in 72% and 85% yields, respectively (Scheme 2). The high yield of **1** (i.e., >50%) obtained when using stoichiometric I_2_ is noteworthy, as a statistical mixture of tetraallylsilane, **1**, and **2** may have been anticipated. Indeed, compared with other reported methods for diallylsilane rearrangements (e.g., acid-promoted [4,5]), only the use of iodine was found to provide good yields of mono- vs. di-rearranged products with limited byproduct formation (e.g., deallylation) in reactions with tetraallylsilane. We attribute the selectivity in part to the reduced nucleophilicity of the initially formed iodosilane intermediate **Si-I**, allowing for the selective consumption of tetraallylsilane over **Si-I**.

**Scheme 3:**
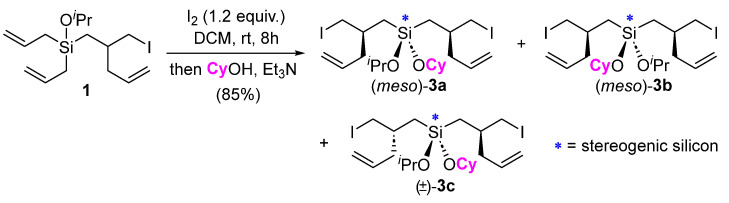
Further reaction of iodine rearrangement product **1** and the generation of **3**, featuring two different alkoxy ligands on silicon. All three diastereomers contain a stereogenic silicon atom, according to the IUPAC definition, where the interchange of any two groups leads to a stereoisomer. For instance, interchanging the alkoxy groups in **3a** leads to its diastereomer **3b**, and a switch of the alkoxy groups on **3c** produces its enantiomer.

Compound **1**, being a diallylsilane, could also be reengaged in an iodine-promoted rearrangement. Performing this type of sequential rearrangement allows for the incorporation of different alcohols for subsequent etherification. For instance, treatment of **1** with I_2_ followed by cyclohexanol gave compound **3**, featuring two different alkoxy ligands attached to silicon (Scheme 3). This strategy might prove useful for reactions involving two different silicon-tethered alcohols where the use of a dichlorosilane for this purpose is challenging (e.g., due to homocoupling) [6,7]. There are three possible diastereomers of compound **3** (i.e., **3a**–**c**) that we were unable to separate with flash column chromatography on silica. The ^1^H NMR signals for these diastereomers were overlapping and/or coincident; however, some signals were resolved by ^13^C NMR, making it clear that all three diastereomers had been obtained (see Supporting Information).

**Scheme 4:**
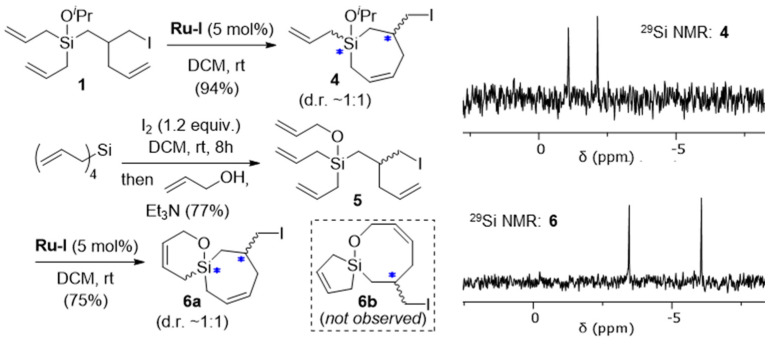
Synthesis of silacycle **4** and oxasilaspirocycle **6a** containing stereogenic carbon and silicon atoms (*) with RCM. ^29^Si NMR data indicate these compounds were obtained as ~1:1 mixtures of diastereomers. The presence of two ^29^Si NMR signals also distinguishes **4** and **6a** from other possible silacyclopentene RCM products (e.g., **6b**).

Each of the diasteromers **3a**–**c** contain a stereogenic silicon atom, according to the IUPAC definition, where the interchange of any two groups attached to silicon leads to a stereoisomer (e.g., from **3a** to **3b** and vice versa) [8]. Compared with the plethora of methods available for the asymmetric synthesis of purely organic molecules, synthetic approaches to silicon-stereogenic organosilanes remain underdeveloped [9,10,11,12,13,14,15,16] despite the potential value of these compounds in biology and material science [17,18,19]. We were inspired, therefore, to consider how this method might be further employed to generate structures containing stereogenic silicon atoms. Building off our previous success with ring-closing metathesis (RCM) reactions of iodine-mediated diallylsilane rearrangement products [20], RCM was pursued as a route to potentially differentiate the olefinic ligands attached to silicon in our rearranged products. In the event, the treatment of a dilute (0.02M) solution of **1** in DCM with Grubbs’ first-generation catalyst (**Ru-I**) at room temperature resulted in its clean conversion to the seven-membered ring silicon-stereogenic silacycle **4** in a high yield (Scheme 4). No evidence for the potential silacyclopentene product was observed with NMR, consistent with Ahmad and coworkers’ observation that silacycloheptene formation is easier than silacyclopentene with RCM [21]. Given the thermodynamic nature of RCM, the results suggests that the ring strain of the seven-membered silacycloalkene is less than that of the five-membered silacycloalkene. Consistent with these results and that of Mahieux et al., who observed selective six-membered ring formation over five-membered ring formation when studying silacycle formation with RCM [22], the RCM of **5** selectively produced the 6,7-spirocycle **6a** rather than the alternative 5,8-spirocyclic isomer **6b**. Compound **6a** was isolated in a 75% yield, with the mass balance appearing to be products arising from cross-metathesis/polymerization, which might also explain the lack of **6b** in the product mixture as this less stable ring system may be susceptible to ring-opening metathesis processes [23,24,25,26]. The ^29^Si NMR spectra of **4** and **6** showed two signals of roughly equal intensity, indicating that a ~1:1 mixture of diastereomers was produced. The presence of two signals in the ^29^Si NMR also provides additional confirmation of their structure and differentiation from the alternative silacyclopentene RCM products (e.g., **6b**), which do not contain a stereogenic silicon atom and would therefore only exhibit a single ^29^Si NMR signal (i.e., a mixture of enantiomers rather than diastereomers would have been produced). Moving forward, different approaches to controlling the relative and absolute stereochemistry of silicon-stereogenic compounds of type **3**, **4**, and **6** will be explored.

## 3. Materials and Methods

### 3.1. General Information

All reactions were carried out in dry vessels under a nitrogen atmosphere at ambient conditions unless otherwise specified. The dry solvents used were prepared by passing the purchased solvents (Thermo Fisher Scientific, Rockford, IL, USA) through a column of activated alumina under nitrogen immediately prior to use. All reagents were purchased from Sigma Aldrich (St. Louis, MI, USA) and used as received unless mentioned otherwise. TLC analysis used 0.25 mm silica layer fluorescence UV_254_ plates (Silicycle, Quebec City, QC, CA). Column chromatography used silica gel (230–400 mesh; Silicycle, Quebec City, QC, CA). For IR, we used a Thermo Fisher Nicolet is10 FT-IR spectrometer equipped with a single-bounce diamond ATR. NMR spectra were recorded on a Bruker 500 MHz spectrometer in CDCl_3_; chemical shifts (d) are given in ppm, and coupling constants (J) are given in Hz. Solvent signals were used as references (CDCl_3_: δ_c_ = 77.0 ppm; residual CHCl_3_ in CDCl_3_: δ_H_ = 7.26 ppm). For HRMS, we used a Bruker Maxis Impact quadrupole time-of-flight LC–MS with electrospray ionization (ESI positive).

### 3.2. Compound Syntheses and Spectral Data

**Diallyl(2-(iodomethyl)pent-4-en-1-yl)(isopropoxy)silane (1).** To a solution of tetraallylsilane (0.19 g, 1.0 mmol) in DCM (10 mL), I_2_ (0.25 g, 1.0 mmol) was added, and the mixture was stirred for 6 h. The solution was then cooled to 0 °C before triethylamine (2.0 mmol) and isopropanol (0.13 mL, 1.5 mmol) were added, and the resulting mixture was stirred and allowed to slowly warm to room temperature over 6 h. The reaction was quenched with water (15 mL) and extracted with DCM (2 × 15 mL). The combined organic extracts were dried over MgSO_4_, filtered, and concentrated on a rotary evaporator. The crude product was purified with column chromatography on silica (20:1 to 10:1 Hexanes:EtOAc), giving **1** (0.27 g, 72%) as an oil. IR (ATR) 3069, 2970, 2911, 1639, 1589, 1428, 1381, 1368, 1219, 1109, 1021, 997, 914, 877, 732 cm^−1^. ^1^H NMR (500 MHz, CDCl_3_) δ 5.89–5.76 (m, 2H), 5.70 (ddt, J = 17.2, 10.1, 7.2 Hz, 1H), 5.13 (dd, J = 17.2, 1.8 Hz, 1H), 5.10–5.06 (m, 1H), 4.98–4.89 (m, 4H), 4.08 (dt, J = 12.1, 6.0 Hz, 1H), 3.36 (dd, J = 9.5, 4.3 Hz, 1H), 3.28 (dd, J = 9.5, 5.4 Hz, 1H), 2.21 (dddt, J = 13.7, 6.8, 5.5, 1.4 Hz, 1H), 2.16–2.03 (m, 1H), 1.75–1.65 (m, 3H), 1.59 (dddd, J = 12.6, 6.9, 3.5, 1.4 Hz, 1H), 1.33–1.23 (m, 1H), 1.16 (d, J = 6.0 Hz, 7H), 0.71 (dd, J = 6.8, 4.1 Hz, 1H). ^13^C NMR (126 MHz, CDCl_3_) δ 135.61, 133.49, 117.29, 114.46, 114.44, 65.55, 41.04, 34.68, 30.90, 25.83, 22.47, 22.43, 19.27, 19.17. HRMS (ESI+) Calcd for C_15_H_27_INaOSi (M + Na): 401.0774. Found: 401.0773.

**Bis(2-(iodomethyl)pent-4-en-1-yl)diisopropoxysilane (2).** To a solution of tetraallylsilane (0.19 g, 1.0 mmol) in DCM (10 mL), I_2_ (0.76 g, 3.0 mmol) was added, and the mixture was stirred for 6 h. The solution was then cooled to 0 °C before triethylamine (3.5 mmol) and isopropanol (0.21 mL, 2.5 mmol) were added, and the resulting mixture was stirred and allowed to slowly warm to room temperature over 6 h. The reaction was quenched with water (15 mL) and extracted with DCM (2 × 15 mL). The combined organic extracts were dried over MgSO_4_, filtered, and concentrated on a rotary evaporator. The crude product was purified with column chromatography on silica (20:1 to 10:1 Hexanes:EtOAc), giving **2** (0.42 g, 85%) as an oil and ~1:1 mixture of diastereomers. Spectral data for the mixture of two diastereomers: IR (ATR) 3072, 2975, 1638, 1589, 1428, 1380, 1219, 1109, 1021, 997, 915, 880 cm^−1^. ^1^H NMR (500 MHz, CDCl_3_) δ 5.71 (ddtd, J = 17.1, 10.0, 7.2, 2.5 Hz, 4H), 5.13 (dq, J = 17.1, 1.7 Hz, 4H), 5.10–5.06 (m, 4H), 4.27–4.09 (m, 4H), 3.43–3.31 (m, 8H), 2.22 (dtdd, J = 12.5, 5.6, 2.6, 1.3 Hz, 4H), 2.11 (ddddd, J = 15.1, 7.4, 6.3, 2.5, 1.2 Hz, 4H), 1.59–1.51 (m, 4H), 1.29–1.15 (m, 28H), 0.68 (dd, J = 6.7, 1.4 Hz, 4H). ^13^C NMR (126 MHz, CDCl_3_) δ 137.91, 115.51, 64.77, 44.48, 28.60, 25.56, 22.33, 19.74. HRMS (ESI+) Calcd for C_18_H_34_I_2_NaO_2_Si (M + Na): 587.0315. Found: 587.0312.

**(Cyclohexyloxy)bis(2-(iodomethyl)pent-4-en-1-yl)(isopropoxy)silane (3)**. To a solution of **1** (0.1 g, 0.26 mmol) in DCM (2.6 mL), iodine (0.078 g, 1.2 equiv.) was added, and the mixture was stirred for 6 h before cooling to 0 °C and triethylamine (0.09 mL, 2.5 equiv.) and cyclohexanol (0.04 mL, 1.5 equiv.) were added. The resulting mixture was allowed to slowly warm to room temperature before quenching with water (15 mL) and extracting with DCM (2 × 15 mL). The combined organic extracts were dried over MgSO_4_, filtered, and concentrated on a rotary evaporator. The crude product was purified with column chromatography on silica (20:1 to 10:1 Hexanes:EtOAc), giving **3** (0.13 g, 85%) as an oil and mixture of diastereomers. Spectral data for the mixture of three diastereomers: IR (ATR) 3068, 2972, 2951, 1610, 1569, 1418, 1410, 1353, 1311, 1201, 1109, 1021, 997, 914, 877, 732 cm^−1^. ^1^H NMR (500 MHz, CDCl_3_) δ 5.88–5.77 (m, 2H), 5.72 (dqt, J = 17.2, 7.3, 2.6 Hz, 2H), 5.17–5.04 (m, 4H), 4.99–4.88 (m, 4H), 4.26–4.13 (m, 2H), 3.85–3.74 (m, 2H), 3.44–3.30 (m, 8H), 2.22 (dttd, J = 13.9, 5.6, 2.7, 1.4 Hz, 4H), 2.11 (dtdd, J = 13.9, 7.4, 2.6, 1.3 Hz, 4H), 1.81–1.77 (m, 4H), 1.75–1.69 (m, 4H), 1.63–1.47 (m, 8H), 1.40–1.24 (m, 10H), 1.21–1.16 (m, 14H), 0.71–0.66 (m, 4H). ^13^C NMR (126 MHz, CDCl_3_) δ ^13^C NMR (126 MHz, CDCl_3_) δ 135.78, 135.67, 133.48, 117.29, 117.12, 114.39, 70.70, 65.08, 65.05, 65.03, 41.14, 41.12, 41.10, 41.07, 41.00, 40.96, 35.71, 34.75, 34.71, 34.57, 25.81, 25.72, 25.50, 23.92, 23.90, 22.41, 22.39, 19.79, 19.75, 19.70, 19.35, 19.33, 18.82, 18.80. HRMS (ESI+) Calcd for C_21_H_38_I_2_NaO_2_Si (M + Na): 627.0628. Found: 627.0631.

**1-Allyl-3-(iodomethyl)-1-isopropoxy-2,3,4,7-tetrahydro-1H-silepine (4).** To a solution of **1** (0.19 g, 0.5 mmol) in degassed DCM (25 mL), **Ru-I** (0.02 g, 0.025 mmol) was added, and the mixture was stirred for 15 h. The solution was then concentrated on a rotary evaporator, and the crude product was purified with chromatography on silica (20:1 to 10:1 Hexanes:MTBE) giving **4** (0.165 g, 94%) as an oil and ~1:1 mixture of diastereomers. Spectral data for the mixture of two diastereomers: IR (ATR) 3042, 2972, 2911, 1614, 1562, 1435, 1410, 1364, 1207, 1151, 1009, 998, 901, 876, 731 cm^−1^. ^1^H NMR (500 MHz, CDCl_3_) δ 5.87–5.66 (m, 4H), 5.65–5.55 (m, 2H), 4.93 (dq, J = 6.3, 1.6 Hz, 1H), 4.91–4.87 (m, 3H), 4.03 (dq, J = 12.0, 6.0 Hz, 2H), 3.21 (dd, J = 5.5, 2.3 Hz, 1H), 3.19 (dd, J = 5.4, 2.2 Hz, 1H), 3.14 (dd, J = 9.5, 7.1 Hz, 2H), 2.29 (ddt, J = 14.2, 7.9, 2.1 Hz, 1H), 2.19 (ddt, J = 13.8, 8.1, 2.1 Hz, 1H), 2.09 (dtd, J = 13.8, 8.2, 1.4 Hz, 2H), 1.93 (ddtt, J = 12.6, 7.3, 3.3, 2.0 Hz, 1H), 1.88–1.75 (m, 2H), 1.72–1.63 (m, 3H), 1.62–1.53 (m, 4H), 1.23–1.17 (m, 2H), 1.16 (d, J = 5.9 Hz, 6H), 1.15 (d, J = 5.9 Hz, 6H), 0.76 (dd, J = 14.2, 11.3 Hz, 2H), 0.69 (dd, J = 14.6, 11.0 Hz, 2H). ^13^C NMR (126 MHz, CDCl_3_) δ 133.76, 133.56, 127.63, 126.88, 126.69, 126.52, 113.97, 113.87, 65.60, 65.36, 38.58, 36.58, 34.40, 34.21, 25.80, 25.78, 25.71, 24.34, 23.83, 23.48, 21.42, 20.06, 19.94, 14.87, 14.79. HRMS (ESI+) Calcd for C_13_H_24_IOSi (M + H) 351.0641. Found 351.0641.

**Diallyl(allyloxy)(2-(iodomethyl)pent-4-en-1-yl)silane (5).** To a solution of tetraallylsilane (0.2 g, 1.0 mmol) in DCM (10 mL), I_2_ (0.26 g, 1.0 mmol) was added, and the mixture was stirred for 6 h. The solution was then cooled to 0 °C before diisopropylethylamine (0.45 mL, 2.5 mmol) and allyl alcohol (0.11 mL, 1.5 mmol) were added, and the resulting mixture was stirred and allowed to slowly warm to room temperature over 6 h. The reaction was quenched with water (15 mL) and extracted with DCM (2 × 15 mL). The combined organic extracts were dried over MgSO_4_, filtered, and concentrated on a rotary evaporator. The crude product was purified with column chromatography on silica (20:1 to 10:1 Hexanes:EtOAc), giving **5** (0.29 g, 77%) as an oil. IR (ATR) 3076, 2972, 2909, 1629, 1418, 1219, 1157, 1031, 991, 826, 787 cm^−1^. ^1^H NMR (500 MHz, CDCl_3_) δ 5.91 (ddt, J = 17.1, 10.4, 4.7 Hz, 1H), 5.82 (ddtd, J = 16.9, 10.1, 8.0, 5.3 Hz, 2H), 5.74–5.65 (m, 1H), 5.27 (dq, J = 17.1, 1.8 Hz, 1H), 5.17–5.05 (m, 3H), 5.00–4.91 (m, 4H), 4.23 (dq, J = 4.8, 1.7 Hz, 2H), 3.34 (dd, J = 9.7, 4.3 Hz, 1H), 3.29 (dd, J = 9.6, 5.2 Hz, 1H), 2.20 (dddt, J = 13.7, 6.9, 5.4, 1.4 Hz, 1H), 2.10 (dtt, J = 13.9, 7.4, 1.1 Hz, 1H), 1.80–1.66 (m, 4H), 1.60 (dddd, J = 11.6, 6.8, 5.3, 1.7 Hz, 1H), 0.77 (d, J = 6.8 Hz, 2H). ^13^C NMR (126 MHz, CDCl_3_) δ 136.66, 135.48, 133.13, 117.46, 114.74, 114.73, 114.61, 64.26, 41.16, 34.51, 21.82, 21.78, 19.20, 18.95. HRMS (ESI+) Calcd for C_15_H_25_INaOSi (M + Na) 399.0617. Found 399.0618.

**11-(Iodomethyl)-1-oxa-6-silaspiro[5.6]dodeca-3,8-diene (6a).** To a solution of **5** (0.05 g, 0.13 mmol) in degassed DCM (6.5 mL), **Ru-I** (0.008 g, 0.013 mmol) was added, and the mixture was stirred for 15 h. The solution was then concentrated on a rotary evaporator, and the crude product was purified with chromatography on silica (20:1 to 10:1 Hexanes:MTBE) giving **6a** (0.031 g, 75%) as an oil and ~1:1 mixture of diastereomers. Spectral data for the mixture of two diastereomers: IR (ATR) 2955, 2930, 2875, 1460, 1352, 1344, 1254, 1143, 1103, 1004, 969, 835, 774, 741, 724, 666 cm^−1^. ^1^H NMR (500 MHz, CDCl_3_) δ 5.85 (dddd, J = 13.7, 7.5, 5.1, 2.8 Hz, 2H), 5.78 (dt, J = 10.6, 7.3 Hz, 2H), 5.72–5.62 (m, 2H), 5.61–5.56 (m, 2H), 4.41 (dp, J = 5.2, 2.7 Hz, 4H), 3.21 (ddd, J = 9.8, 5.4, 4.6 Hz, 2H), 3.15 (ddd, J = 9.7, 7.2, 2.8 Hz, 2H), 2.32 (ddt, J = 14.3, 7.7, 1.9 Hz, 1H), 2.24–2.08 (m, 4H), 2.06–1.97 (m, 1H), 1.95–1.87 (m, 1H), 1.81 (dddddd, J = 11.1, 9.2, 7.4, 5.6, 3.7, 1.9 Hz, 1H), 1.77–1.63 (m, 2H), 1.40–1.19 (m, 6H), 0.85 (dd, J = 14.2, 11.1 Hz, 1H), 0.79 (dd, J = 14.6, 10.1 Hz, 1H). ^13^C NMR (126 MHz, CDCl_3_) δ 128.74, 128.70, 128.29, 127.17, 126.40, 126.03, 123.71, 123.48, 63.32, 63.30, 39.11, 36.72, 34.27, 33.97, 25.08, 24.62, 19.38, 19.34, 15.54, 15.40, 11.22, 9.58. HRMS (ESI+) Calcd for C_11_H_17_INaOSi (M + Na) 342.9991. Found 342.9990.

## Data Availability

The original contributions presented in the study are included in the article/supplementary material; further inquiries can be directed to the corresponding author.

## Supplementary Materials

The following supporting information can be downloaded at: https://www.mdpi.com/article/10.3390/ijms25189996/s1.
