# Supplementary material for: Iodine Rearrangements of Tetraallylsilane and Synthesis of Silicon-Stereogenic Organosilanes
Source: Int J Mol Sci. 2024 Sep 17;25(18):9996. doi: 10.3390/ijms25189996 (PMC11432033; doi:10.3390/ijms25189996)

Supporting Information for

Iodine Rearrangements of Tetraallylsilane and Synthesis of Silicon-Stereogenic Organosilanes

Elliott D. Tan, Kerry E. Wier, and Gregory W. O'Neil*
Department of Chemistry, Western Washington University, Bellingham, WA 98229 (USA)
*Corresponding author. Email: oneilg@wwu.edu

Contents:

NMR Spectra for Compound:	Page
Diallyl(2-(iodomethyl)pent-4-en-1-yl)(isopropoxy)silane (**1**)	S2
Bis(2-(iodomethyl)pent-4-en-1-yl)diisopropoxysilane (**2**)	S3
(Cyclohexyloxy)bis(2-(iodomethyl)pent-4-en-1-yl)(isopropoxy)silane (**3**)	S4
1-Allyl-3-(iodomethyl)-1-isopropoxy-2,3,4,7-tetrahydro-1H-silepine (**4**)	S5-6
Diallyl(allyloxy)(2-(iodomethyl)pent-4-en-1-yl)silane (**5**)	S7
11-(Iodomethyl)-1-oxa-6-silaspiro[5.6]dodeca-3,8-diene (**6**)	S8-9

^{1}H NMR spectrum ($CDCl_3$, 500 MHz)

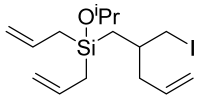

1

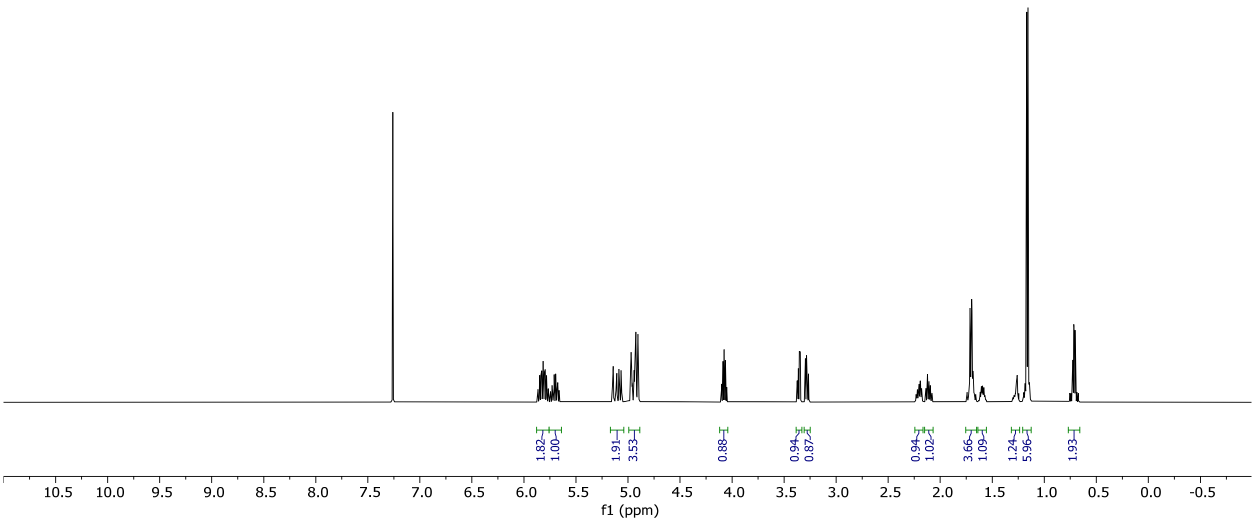

^{13}C{^{1}H} NMR spectrum ($CDCl_3$, 126 MHz)

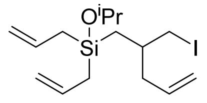

1

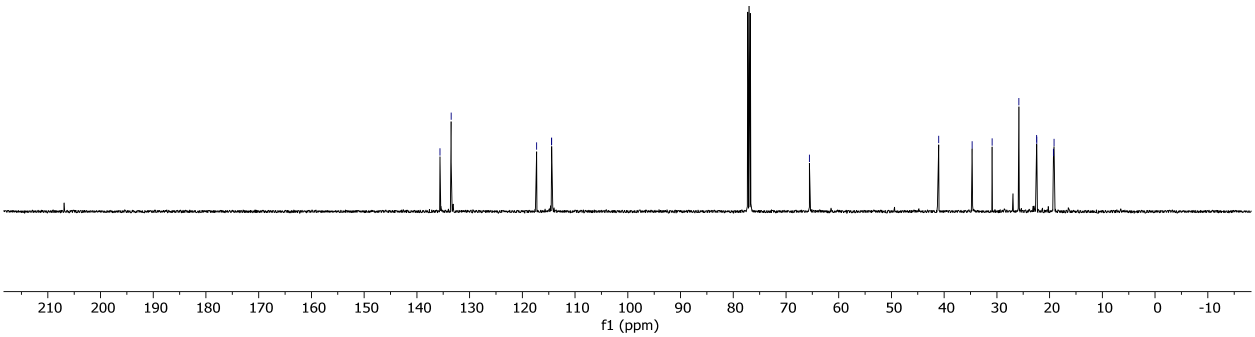

^{1}H NMR spectrum ($CDCl_3$, 500 MHz)

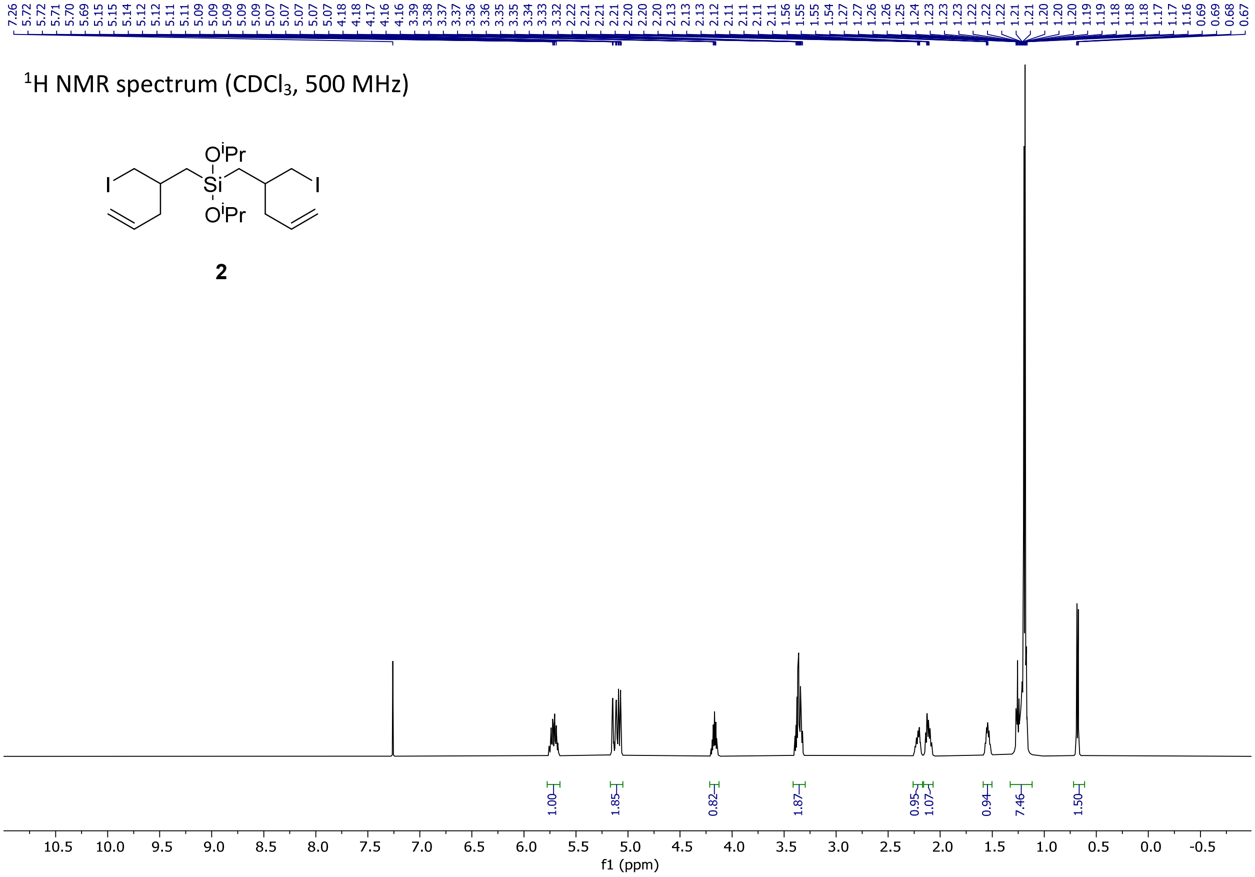

^{13}C{^{1}H} NMR spectrum ($CDCl_3$, 126 MHz)

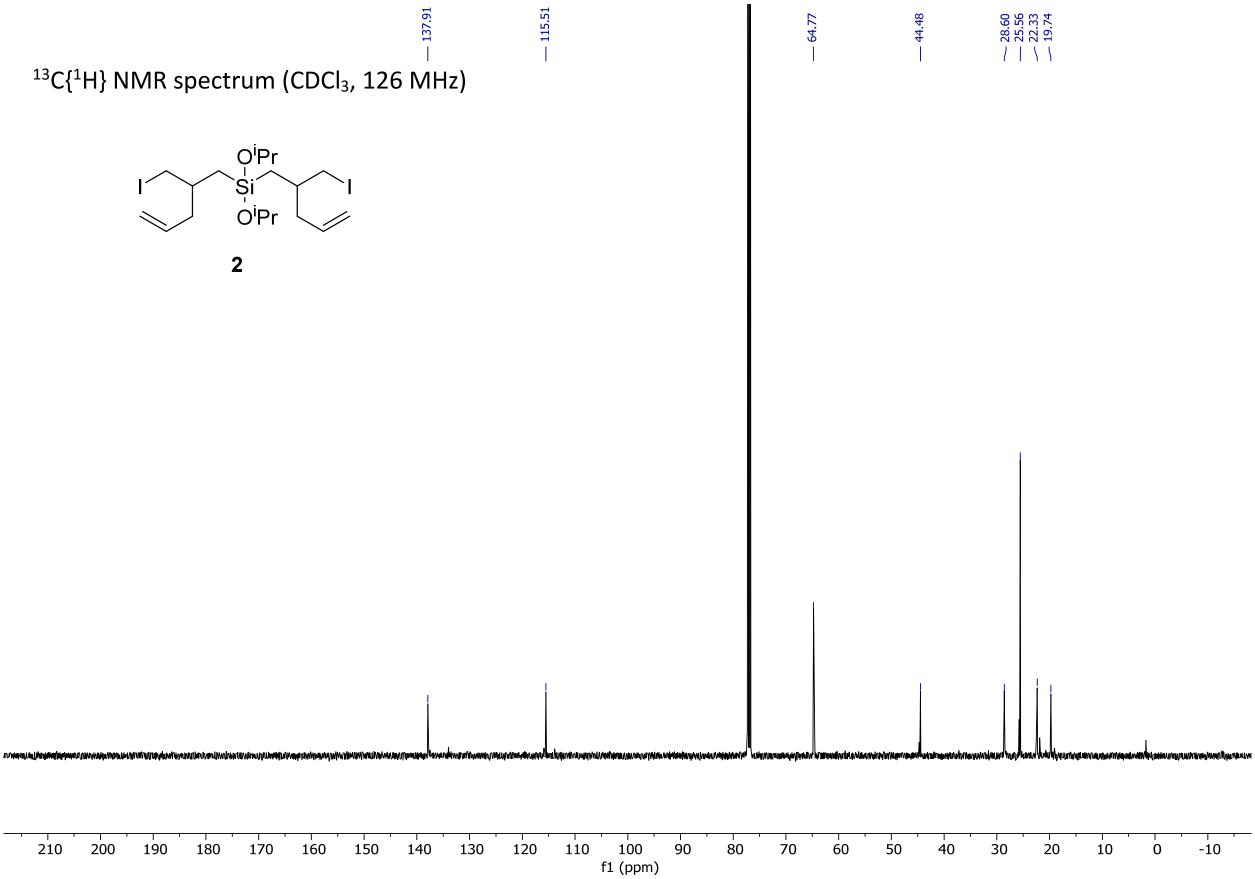

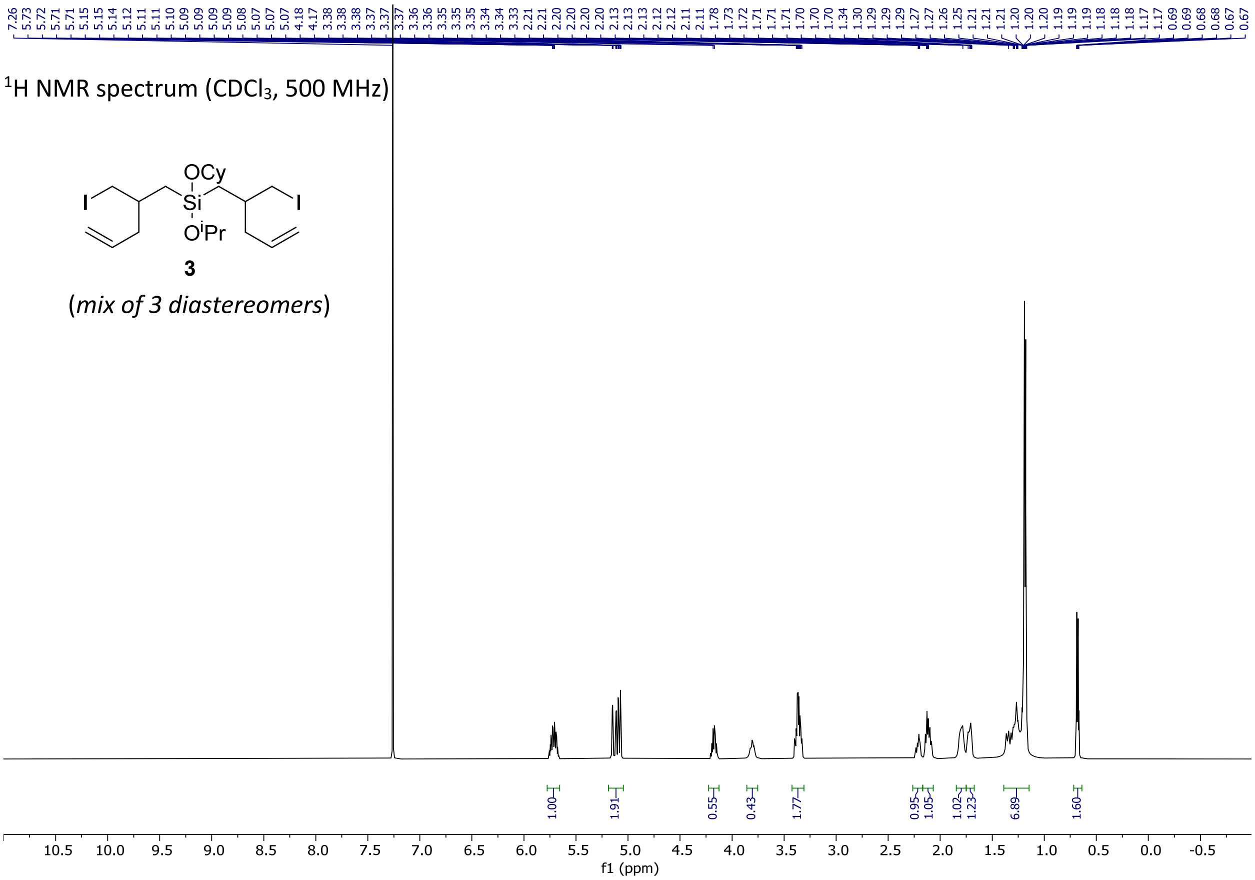
7.26 5.73 5.72 5.71 5.71 5.15 5.15 5.14 5.12 5.11 5.11 5.10 5.09 5.09 5.09 5.09 5.08 5.07 5.07 5.07 4.18 4.17 3.38 3.38 3.38 3.38 3.37 3.37 3.36 3.36 3.35 3.35 3.35 3.34 3.34 3.33 2.21 2.21 2.20 2.20 2.20 2.20 2.13 2.13 2.13 2.12 2.12 2.11 1.78 1.73 1.72 1.71 1.71 1.71 1.70 1.70 1.70 1.34 1.30 1.29 1.29 1.29 1.27 1.27 1.26 1.25 1.21 1.21 1.21 1.20 1.20 1.20 1.19 1.19 1.19 1.18 1.18 1.18 1.17 0.69 0.69 0.68 0.67 0.67
^{1}H NMR spectrum (CDCl$_3$, 500 MHz)
OCy
I
Si
I
O^{i}Pr
3
(*mix of 3 diastereomers*)
1.00 1.91 0.55 0.43 1.77 0.95 1.05 1.02 1.23 6.89 1.60
10.5 10.0 9.5 9.0 8.5 8.0 7.5 7.0 6.5 6.0 5.5 5.0 4.5 4.0 3.5 3.0 2.5 2.0 1.5 1.0 0.5 0.0 -0.5
f1 (ppm)

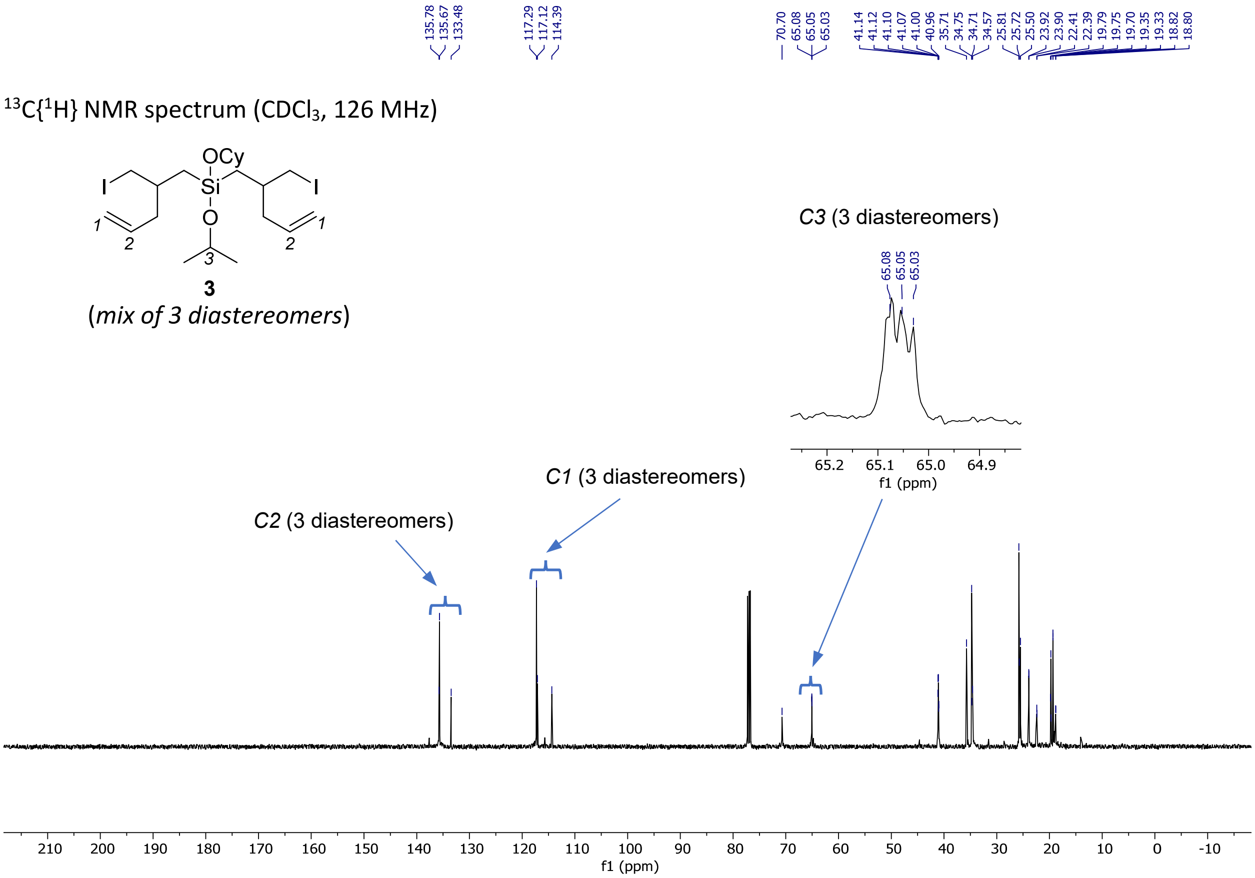
135.78 135.67 133.48 117.29 117.12 114.39 70.70 65.08 65.05 65.03 41.14 41.12 41.10 41.07 41.00 40.96 35.71 34.75 34.71 34.57 25.81 25.72 25.50 23.92 23.90 22.41 22.39 19.79 19.75 19.70 19.35 19.33 18.82 18.80
^{13}C{^{1}H} NMR spectrum (CDCl$_3$, 126 MHz)
OCy
I
Si
I
O
1
2
3
2
1
3
(*mix of 3 diastereomers*)
C3 (3 diastereomers)
65.08 65.05 65.03
65.2 65.1 65.0 64.9
f1 (ppm)
C1 (3 diastereomers)
C2 (3 diastereomers)
210 200 190 180 170 160 150 140 130 120 110 100 90 80 70 60 50 40 30 20 10 0 -10
f1 (ppm)

^{1}H NMR spectrum ($CDCl_3$, 500 MHz)

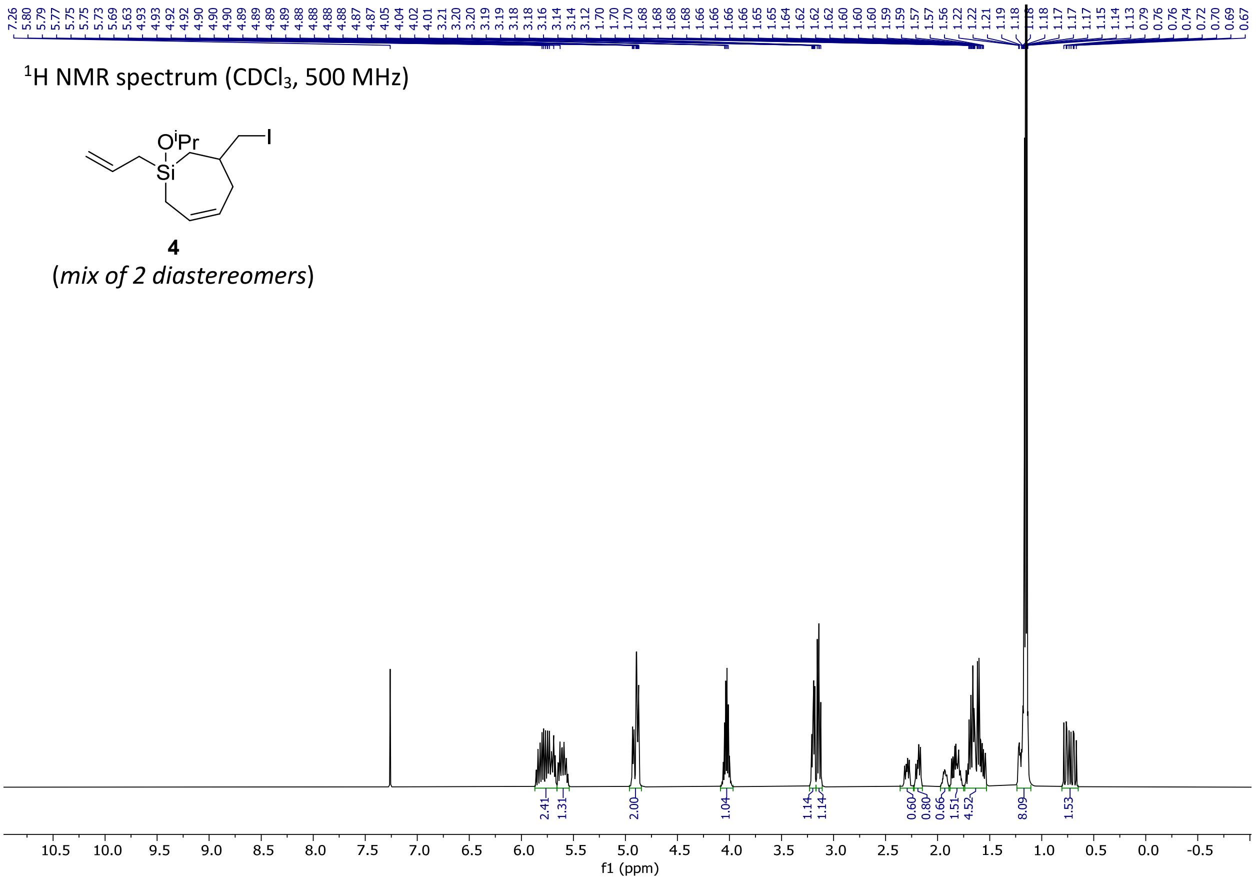

COSY spectrum ($CDCl_3$, 500 MHz)

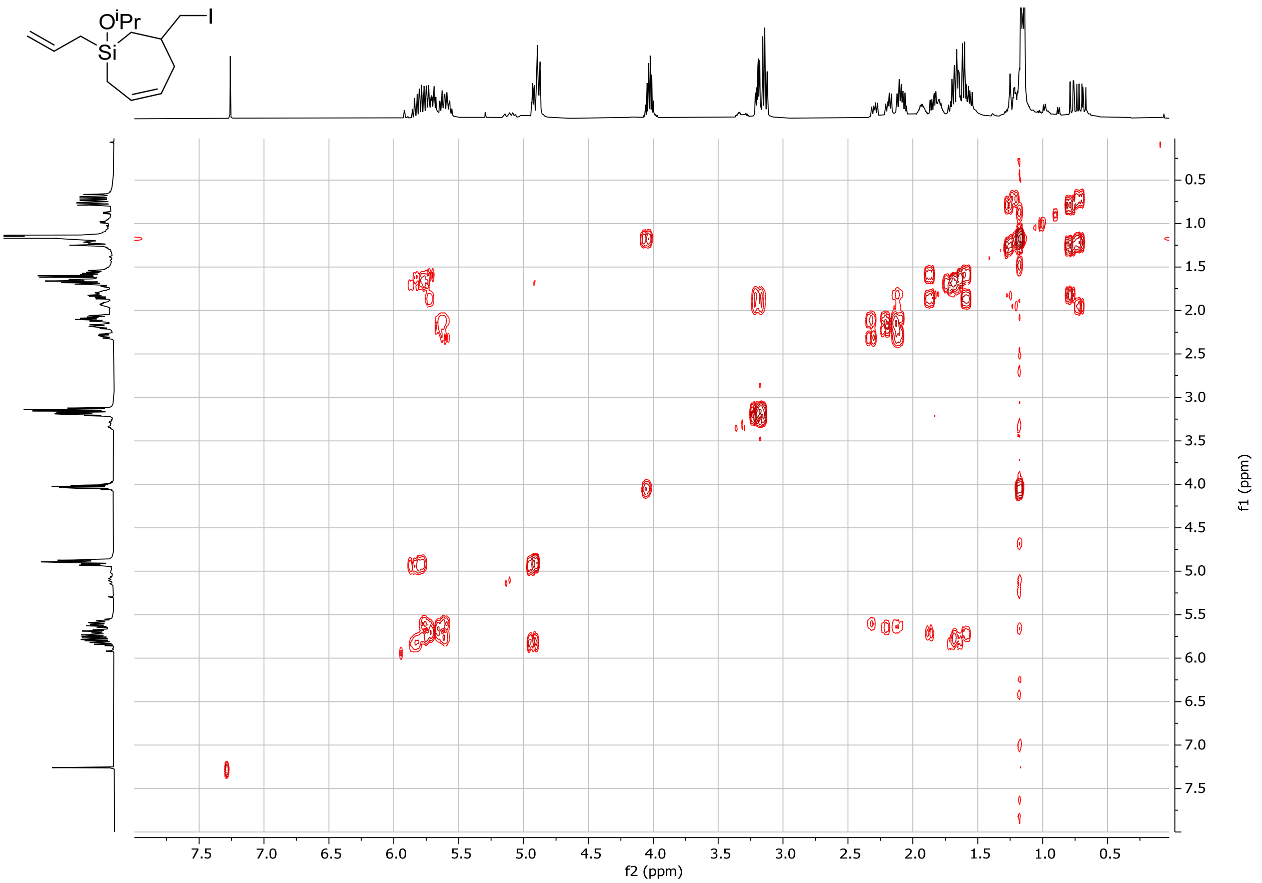

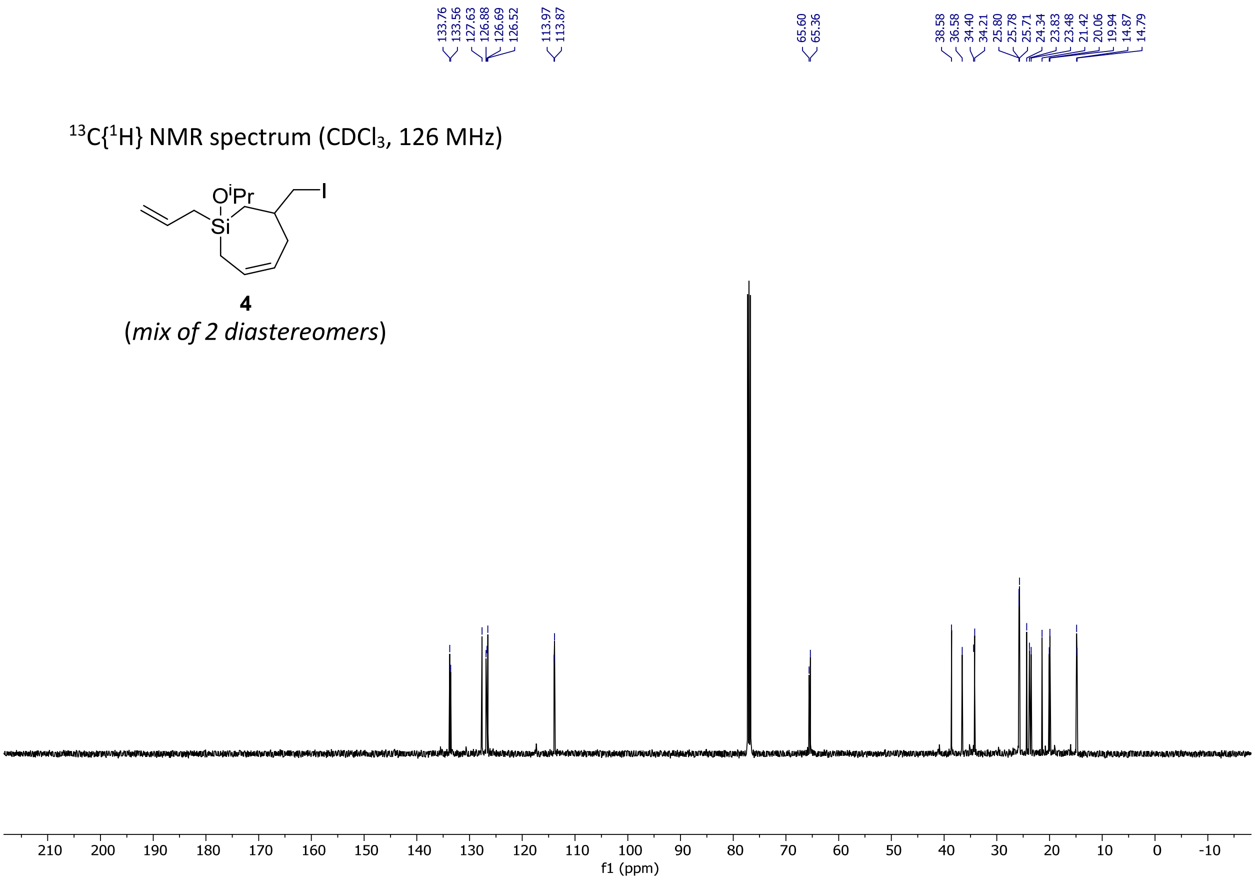
133.76
133.56
127.63
126.88
126.69
126.52
113.97
113.87
65.60
65.36
38.58
36.58
34.40
34.21
25.80
25.78
25.71
24.34
23.83
23.48
21.42
20.06
19.94
14.87
14.79
$^{13}C\{^{1}H\}$ NMR spectrum ($CDCl_3$, 126 MHz)
O^{i}Pr
Si
I
4
(*mix of 2 diastereomers*)
210 200 190 180 170 160 150 140 130 120 110 100 90 80 70 60 50 40 30 20 10 0 -10
f1 (ppm)

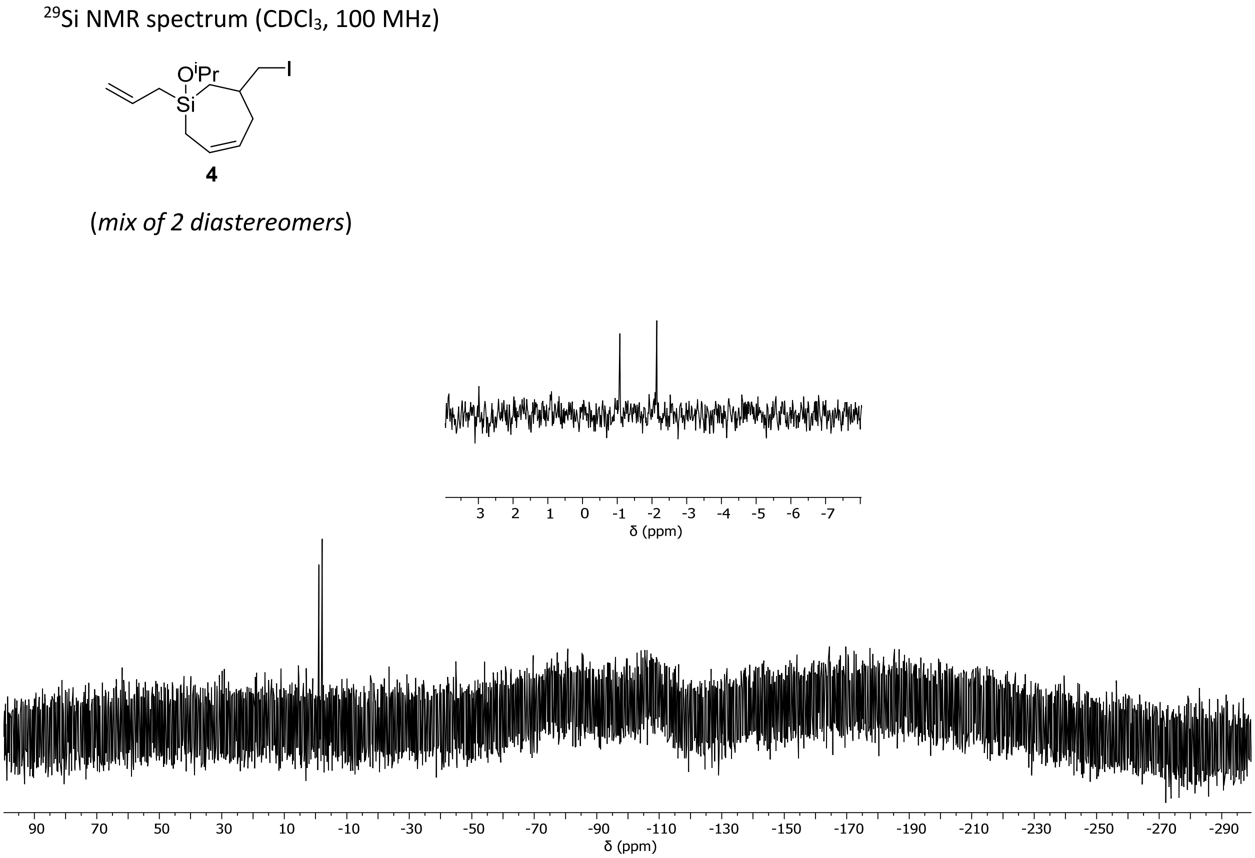
^{29}Si NMR spectrum ($CDCl_3$, 100 MHz)
O^{i}Pr
Si
I
4
(*mix of 2 diastereomers*)
3 2 1 0 -1 -2 -3 -4 -5 -6 -7
δ (ppm)
90 70 50 30 10 -10 -30 -50 -70 -90 -110 -130 -150 -170 -190 -210 -230 -250 -270 -290
δ (ppm)

^{1}H NMR spectrum ($CDCl_3$, 500 MHz)

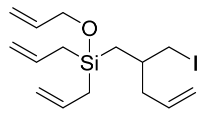

5

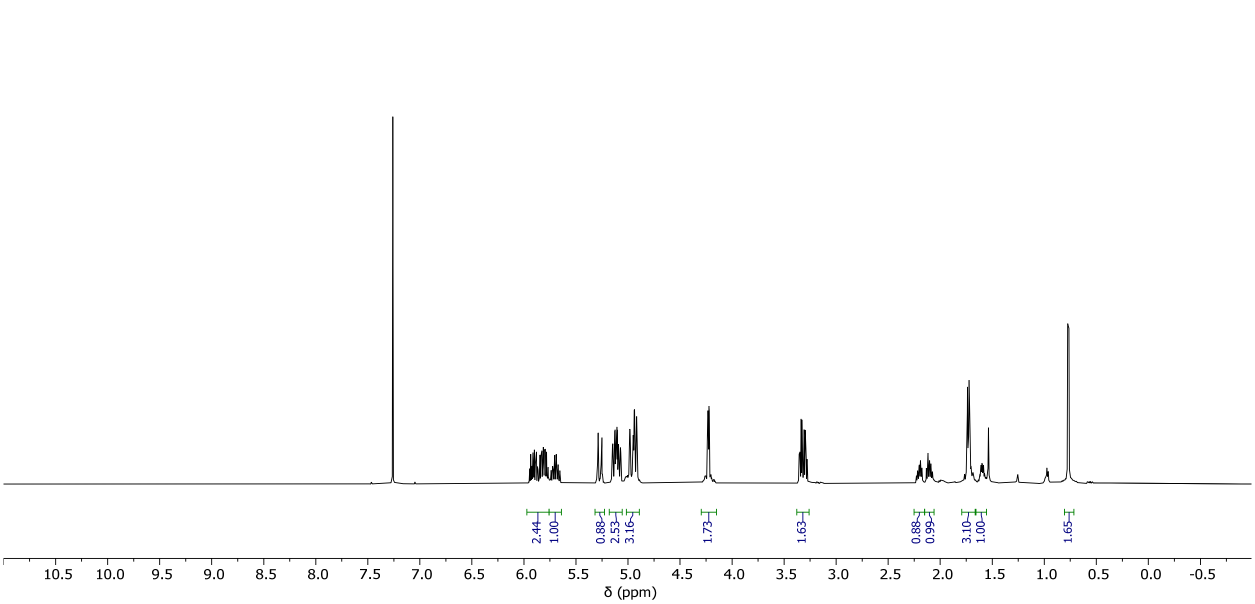

$^{13}C\{^{1}H\}$ NMR spectrum ($CDCl_3$, 126 MHz)

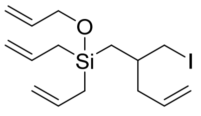

5

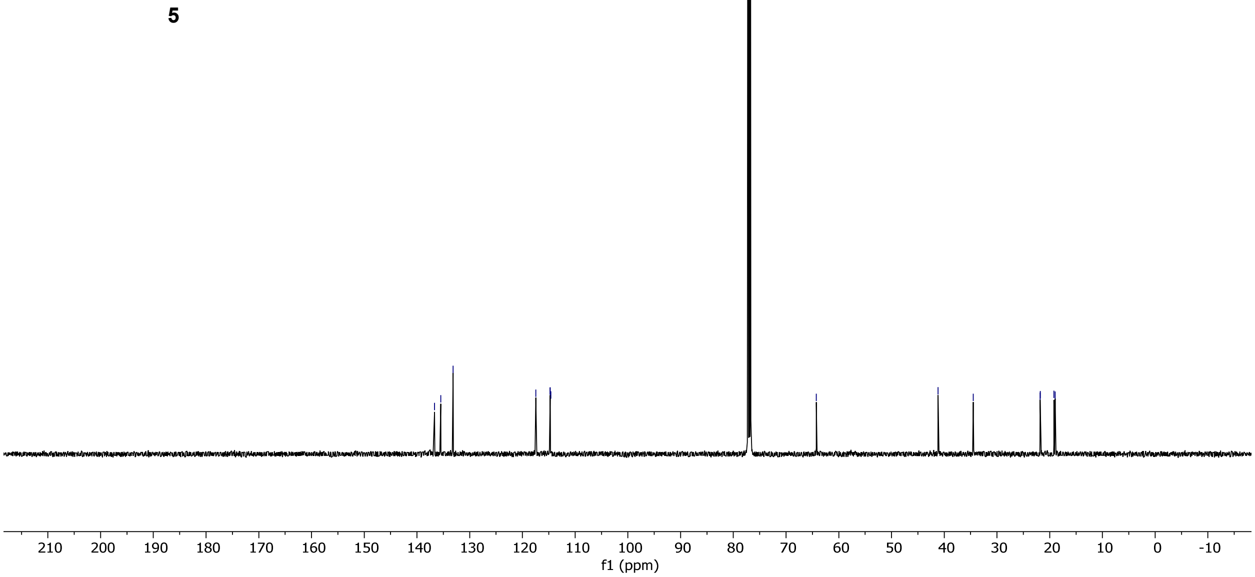

^{1}H NMR spectrum ($CDCl_3$, 500 MHz)

6a

(*mix of 2 diastereomers*)

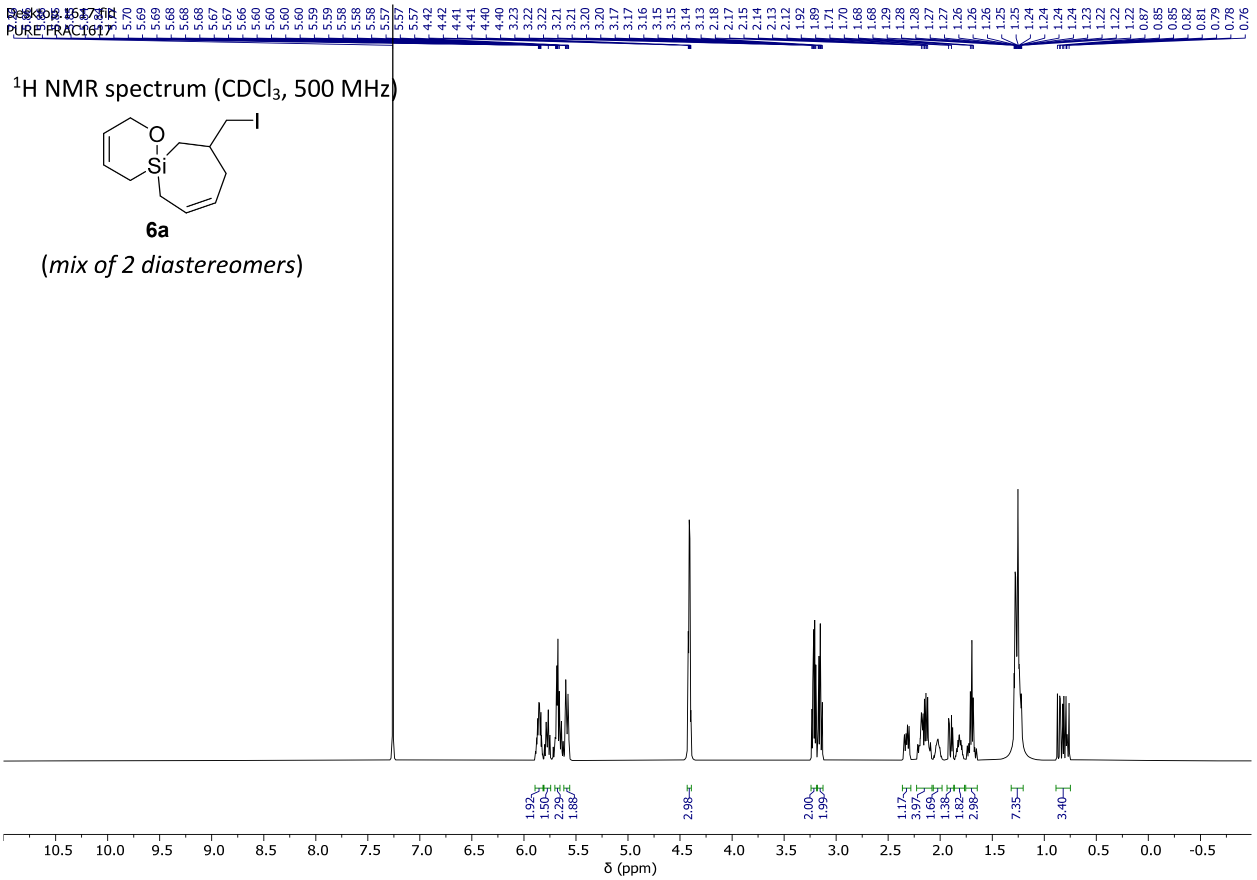

COSY spectrum ($CDCl_3$, 500 MHz)

6a

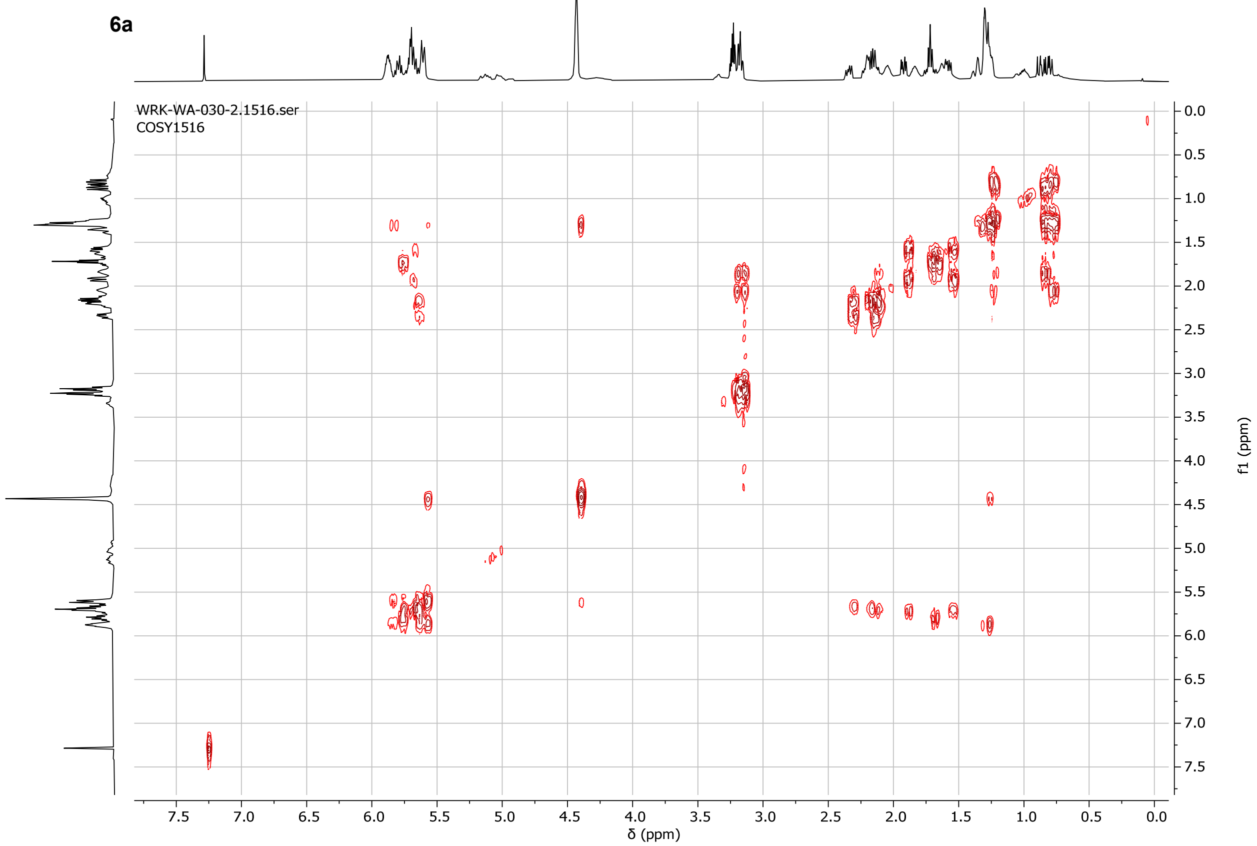

WRK-WA-030-3.1516.fid
13C

$^{13}C\{^{1}H\}$ NMR spectrum ($CDCl_3$, 126 MHz)

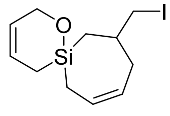

6a

(*mix of 2 diastereomers*)

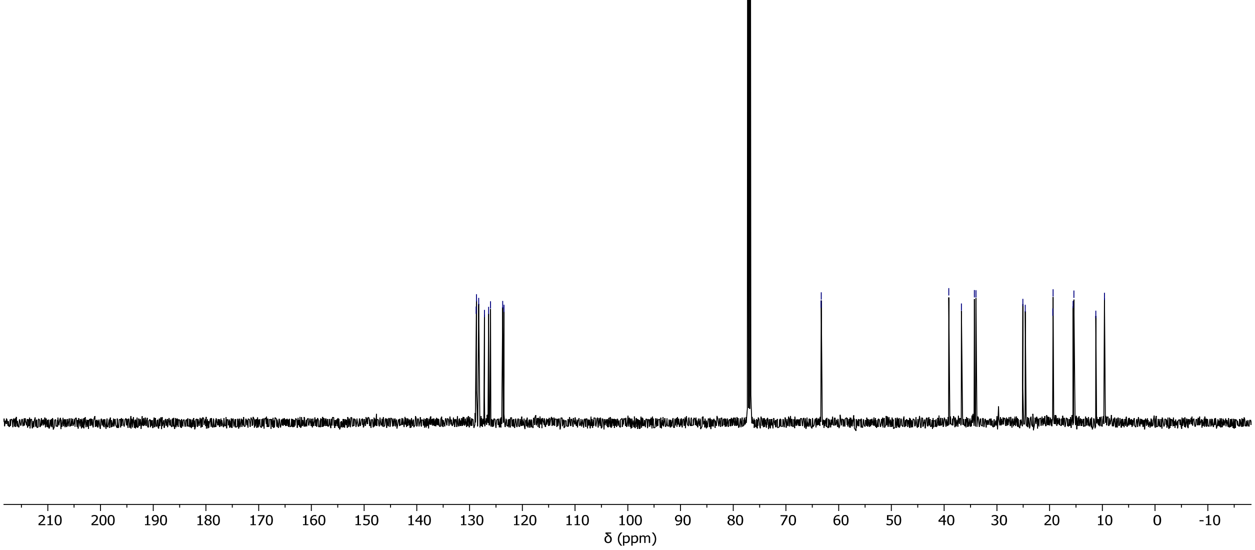

^{29}Si NMR spectrum ($CDCl_3$, 100 MHz)

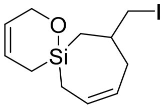

6a

(*mix of 2 diastereomers*)

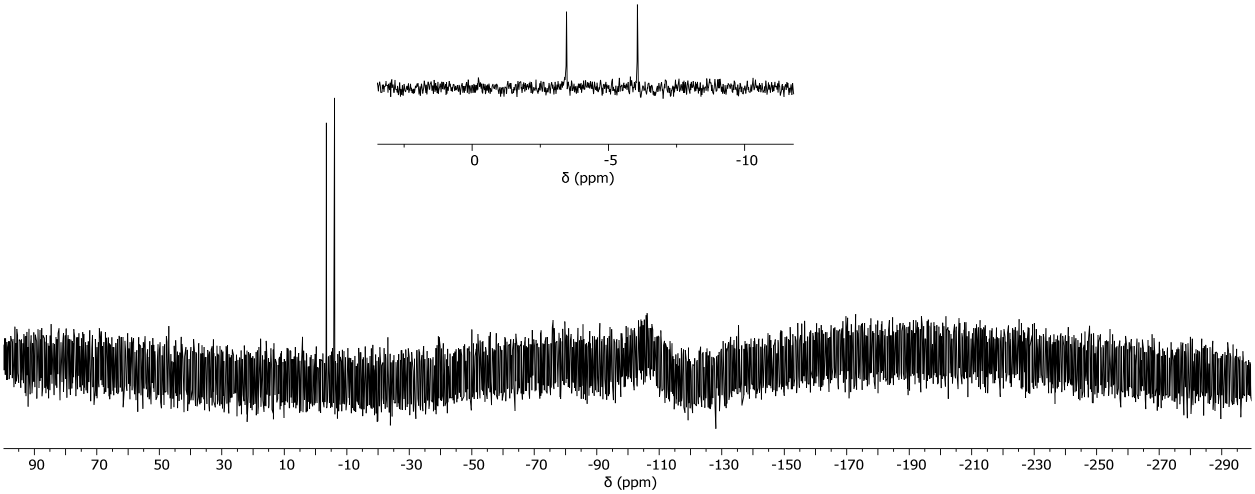

Supplement: Supplementary file 1 [file ijms-25-09996-s001.zip › ijms-3182002-supplementary.pdf]
